# Suicide among Health Care Professionals—An Indian Perspective

**DOI:** 10.3390/healthcare10020354

**Published:** 2022-02-11

**Authors:** Nishant Das, Prerna Khar, Sagar Karia, Nilesh Shah

**Affiliations:** 1 Department of Psychiatry, Lokmanya Tilak Municipal Medical College and General Hospital, Sion, Mumbai 400022, India; nishant.das16@gmail.com (N.D.); drnilshah@hotmail.com (N.S.); 2 Department of Psychiatry, TNMC and BYL Nair Charitable Hospital, Mumbai 400008, India; prernakhar@gmail.com

**Keywords:** suicide, health care provider, prevention

## Abstract

Suicide is a global phenomenon that claims a person’s life every 40 s. The suicide-mortality rate in India is higher than the worldwide average for health care professionals (HCP). The treatment gap for mental health care is alarming, more than 80% in India which has improved compared to a decade. Among the methods chosen by HCPs for dying by suicide, violent suicide methods are more common. Hanging is the most common means, followed by lethal injection and jumping from a building. Among the medical students and professionals in India, academic stress is the leading cause of suicides, followed by mental illness and harassment. Stressfully long working hours, starvation for long hours, inadequate diet, sleep deprivation, inadequate rest, high levels of personal expectations, knowledge of lethal suicide methods, easy access to potentially fatal drugs, apathy, and fearlessness towards death are some of the contributing factors. Primary preventive measures to minimize suicides in HCPs would be to conduct stress-management workshops at an institutional level, routine mental health check-ups in healthcare institutions, mental-health screening for students enrolling into healthcare courses, and prompt referrals to mental healthcare facilities. In addition, telehealth services or mental health services for medical professionals of India are the need of the hour.

## 1. Introduction

“I stepped into this college hoping I would get to learn under such a good institute. However, people started showing their colors… The torture continued to the level that I could not bear. I complained against them but it showed no result… I have lost my professional life, personal life, everything because they have declared that they will not let me learn anything until they are here.” These are a few hard-hitting sentences from the heart-rending suicide note of a postgraduate medical student of a college in an Indian metropolitan city, as reported in a newspaper article [1]. The police found the letter a few hours after the death by suicide. In the past couple of years, the incidence of health care workers dying by suicide has risen.

Society looks up to health care professionals (HCPs), to save their patients’ lives. What then drives a HCP to the brink of suicide? This article aims to explore answers to this disturbing yet essential question.

Suicide is a global phenomenon, yearly taking away lives of nearly 800,000 individuals. As per the World Health Organization (WHO), every 40 s, we lose a person to suicide [2]. The suicide mortality rate in India is 16.3 per 100,000 population (compared to the global average of 10.6) [3]. According to the National Mental Health Survey 2016 (NMHS), the treatment gap for mental health care is alarming, more than 80% in India. This is improving compared to the past decade but the gap remains and this also affects suicidal behavior when it comes to seeking treatment [4].

Health care professionals (HCPs) include doctors, dentists, nurses, ward staff, ancillary staff, and medical/dental students and residents. Studies analyzing suicide among HCPs have mainly focused on doctors/medical students. There is a paucity of literature exploring suicide in nurses/ward staff and ancillary staff, especially in the Indian scenario. We aim to explore the various causes in this diverse group as each sub-group has different risk factors and hence varied preventive strategies. India is home to approximately 1 million doctors, which is the largest globally. The risk of suicide among doctors is considered 2.5 times higher than that in the general population. Some studies have even reported a higher risk, of between five to seven times [5]. In the United States, it is said that yearly, nearly 400 physicians die by suicide [6]. Suicidal ideation among medical students ranges from 1.8% to 53.6%. As per regional studies in India, suicide rates are higher in the south Indian states compared to the northern states [7]. This was also reflected in the study evaluating doctor suicides, by Kishor et al, in which out of the 30 suicides, 57% were from south India [5]. Specialties where a higher rate of suicide is seen are Anesthesiology, General Medicine, and Psychiatry [6].

In the general population, the rate of suicide in men is nearly four times as high as women’s, while among health care professionals (HCPs), the rates are comparable for males and females. In an Australian review analyzing suicide among HCPs during 2001–2012, it was found that the suicide rate among women HCPs was statistically higher than in other professions [8]. Similar findings were also reported in an Indian study by Kirshore et al. where out of the 30 suicides in doctors, 60% were by females [9]. This has been attributed to greater stress perception in women due to balancing their personal and professional lives [8,9].

Leaving aside qualified, practicing professionals, even undergraduate medical and dental aspirants, are at risk. In 2018–2019, out of the student suicides, 10 (55%) occurred in the month in which the results were declared. In 2020, three students died by suicide in the month in which results were reported [9]. This clearly reflects the tremendous pressure of performance.

The COVID-19 pandemic has taken a toll on people of all walks of life, sparing no segment of society. Leaving aside the physical consequences of the disease, the mental health impact of the pandemic and the lockdown has affected society at large. It is estimated that the psychological effects are here to stay. The suicide rates among the general population have seen a rise in the past year either due to financial aspects or mental health concerns. The pandemic has tremendously impacted HCPs. Long working hours, hostile work environment, the frustration of not being able to save lives, isolation from family members, have affected the mental health of HCPs and led to suicide in a few instances [10]. In India, many HCPs have faced a lot of discrimination during the pandemic. There have been instances of violence against doctors due to administrative lapses and landlords refusing to let in those who worked in COVID facilities. This has made many HCPs question their professional choice and has led to a rise in mental health concerns. A retrospective study of media reports of suicide among HCPs, during the first wave of the COVID-19 pandemic showed that worldwide, 26 HCPs had died by suicide. Out of these, eleven were doctors, nine nurses, five paramedics, and one medical student. A majority (14) were females. Most of the deaths were from India (n = 8), followed by the United States (n = 6), and the most common reason was being infected by COVID-19, followed by stressors at the workplace [11].

### Method of Conducting This Review

To identify articles that focused on suicide among healthcare professionals, the terms ‘suicide’, ‘suicidality’, ‘suicide attempts’, ‘suicides’, or ‘healthcare professionals’ and HCPs were used. To identify articles that focused on specific terms, such as ‘causes’, ‘risk factors’, ‘depression’, ‘work stress’, ‘management’, ‘intervention’, ‘factors’, ‘psychosocial issues’ and other terms were used. These two search strategy results were combined with an “and” statement in the following data bases with the time frame being specified from 2000 through 2021. The databases used were Medline, Pubmed and the Cochrane Database on Systematic Reviews. In total, 196 articles were identified which included reviews, mini reviews and clinical studies in healthcare professionals with or at risk for suicide. We included studies with sample sizes of more than 30 participants and that reported either mean scores or percentages with appropriate statistical analysis. All authors reviewed all of the articles and the most relevant ones were chosen for this review. The papers reviewed in this article include articles, clinical studies and research papers on suicide in healthcare professionals. This is supplemented with the personal clinical experience of all the authors in this field who work regularly with this group and have further insight into the problems faced by them.

## 2. Methods Used for Suicide

In an Indian study by Chahal et al., evaluating 358 deaths among HCPs between 2010–2019, it was found that violent suicide methods were more commonly used, with hanging being the most common means of suicide [12]. Kishore et al. explored 30 reported suicides by Indian doctors between the years 2013–2016 and found that 17 doctors (57%) resorted to hanging, 8 (26%) used lethal injections, 5 (17%) jumped from a building, and one jumped in front of a train [5]. This is further supported by a study by Jahan et al., which found that during the COVID-19 pandemic, the most commonly used methods of suicide by HCPs were hanging and jumping from a height [11]. This is in contrast to findings in Australian studies where poisoning has been the most common method. Socio-cultural differences may explain the difference in methods. The apathy and fearlessness towards death may be a possible explanation for choosing more violent means to end one’s life [8].

Socio-cultural differences in methods have also been reflected in an American study done by Yisi et al. exploring suicide among HCPs between 2003–2016. Their study showed that suicide by firearm was the most common method adopted [13]. This may be due to the ease of availability of weapons in the USA.

### 2.1. Etiology and Contributing Factors

A study performed on the worldwide epidemiology, using World Health Organisation (WHO) database, showed that the risk of suicidal behavior was ten times higher in patients having a pre-existing psychiatric disorder than in the general population [14]. The highest increase in mortality caused by unnatural causes in the general population worldwide was attributed to depression (30%), followed by substance-use related disorders (18%), schizophrenia (14%), and personality disorders (13%) [15].

However, among the medical students and professionals in India, it was found that academic stress was the leading cause of suicides, followed by mental illness and harassment [10]. Academic stress was simply the tip of the stress iceberg in HCPs [16]. Stressfully long working hours for graduate interns, postgraduate resident doctors, junior physicians, and nursing professionals significantly impact their mental well-being. They often starve for long hours, consume fast food to save time, have sleep deprivation and are inadequately rested between duty shifts. This significantly leads to burn-out and stress [17].

Additionally, due to the lack of adequate hospital supplies and infrastructure in most districts of India, such professionals have to struggle with providing the best possible care with limited resources [16]. High levels of personal expectations and the resulting frustration of being unable to fulfill them can add to their stress. HCPs are also expected to do research work in a time-limited frame, which is often an underacknowledged source of anxiety and stress among them.

HCPs are not immune to mental health problems. There is often neglect or delay in seeking help regarding mental health among HCPs. The stigma associated with being diagnosed with a mental illness is a cause of the silent suffering of HCPs [18]. Knowledge of the possible adverse drug reactions, apprehension to be on psychiatric medications, and a relatively long course of drugs are some of the reasons for erratic treatment adherence. Due to time constraints and busy work schedules, they often drop out of psychotherapeutic treatment modalities.

Workplace harassment in the form of ragging, discrimination and nepotism has also led to suicides among medical and nursing students and resident trainees. Personal life problems such as marital discord/relationship issues are also some of the prominent causes of suicide among physicians [12].

Various reasons for successful suicides among HCPs include knowledge of lethal methods, easy access to potentially lethal drugs which can cause painless death, apathy, and fearlessness towards death after closely working with life–death situations regularly [12].

### 2.2. Risk Factors and Warning Signs

It is crucial to identify and distinguish risk factors and warning signs of suicide to prevent them in a timely way. While risk factors are empirically derived, enduring and static, warning signs are more proximal and based on clinical assessments. Warning signs represent imminent risk and have more implications for immediate interventions [19]. 

Risk factors: Studies conducted in the last two decades have found the following risk factors of suicide: prior history of self-harm mental illness, anxiety, reaction to stress, personality traits of aggression and impulsivity, substance abuse, depression in primary relatives, gender differences and association to sex, violence, marital problems, financial difficulties, perceived humiliations, family disputes, parental death by suicide, housing insecurity, being married, chronic pain, living alone and break in a steady relationship [3]. While the mentioned risk factors of the general population are pertinent to the subgroup of HCPs, job-related problems, mental illness, physical health problems, substance abuse are the more commonly seen risk factors.

In the ongoing COVID-19 pandemic, a study from the south Indian state of Karnataka found that HCPs with frontline COVID duties (doctors, nurses, and hospital assistants) had higher rates of anxiety and depression as compared to other ancillary medical support staff (pharmacists, radiographers, laboratory technicians). Those HCPs with anxiety and depression reported increased suicidal thoughts [20].

Warning signs: The American Association of Suicidology Working Group proposed the following warning signs of suicide: hopelessness; rage, anger, revenge-seeking behavior; reckless and risky behavior; increasing alcohol or drug use; social withdrawal; feeling trapped; anxiety and agitation; sleep alterations; dramatic mood changes; and no reason for living or no purpose in life [19].

Protective factors of suicide include: absence of risk factors, a history of self-control, cultural or religious beliefs that mitigate against suicide, and fears of harming or letting down family members [19].

### 2.3. Prevention

A good primary preventive measure to minimize suicides in HCP would be to conduct routine mental health check-ups in healthcare institutions and mental-health screening of students enrolling in healthcare courses. Prompt referral to mental health professionals for detailed evaluation may prove vital for those with suicide risk. As confidentiality concerns often demotivate HCPs from seeking mental-health services, it may be worthwhile to have a private partnership of referring them to an institute, different from the parent one. Telepsychiatry may prove to be of immense value in this context. Additionally, stress management workshops or lectures, conducted every 6 or 12 months, may add practical value to the mental healthcare of HCPs at an institutional level.

Documentation of suicide risk: Mental illnesses present with an elevated risk of suicide. Mental-health professionals ought to screen for suicidality without fearing that the interview might induce such thoughts in the patient. Developing a structured suicide risk assessment can help practitioners explore this sensitive yet vital examination area. Asking patients if they have thoughts of killing themselves is a good start to evaluating suicidality. The complete assessment of suicidality includes looking into risk factors, warning signs, and psychosocial stressors. Those who report suicidal ideations should be asked about the plan in detail, including the chance of rescue, preparations for, and rehearsal of the suicide attempt [19]. This will help to plan interventions to reduce the risk of suicide by tackling the various factors.

Interventions: In cases of imminent risk, hospitalization is usually the safest option. However, HCPs often hesitant towards admission to a mental-health facility due the associated stigma. The next best option could be frequent and intensive outpatient treatment with pharmacological and psychotherapeutic interventions. Utilization of the patient’s support systems (family members, friends, or co-workers) is essential. Caregivers are to be educated about the risk of suicide, preventive measures such as keeping people under close watch and removing potentially lethal objects and medications from the vicinity. An action plan can be formed with their collaboration in case the patient becomes acutely suicidal [19].

Another common strategy is developing a crisis plan, which contains a detailed list of steps for the patient to follow when they are suicidal. This comprises pharmacological interventions, behavioral interventions and contacting significant others and a mental-health professional. Reporting to a local emergency department is the final step in the crisis care plan. Giving the patient a written algorithm to follow during times of increased distress will help improve their available options [19].

Recommendations: There are various websites and telehealth services such as www.drs4drs.com.au for medical professionals and students of Australia [21]. Similar telehealth services dedicated to medical professionals of India are the need of the hour. KIRAN, a 24/7 national helpline has been set up by the Ministry of Social Justice, which can be reached at 1800-599-0019 [22]. HCPs can currently avail psychological help options available for the general public, such as Suicide Prevention India Foundation (spif.in), which has teleconsultation options and suicide-gatekeeper training programs. Other psychological helplines available for the general public are iCall by Tata Institute of Social Sciences, helplines by Mumbai-based organizations such as Aasra and Samaritans Mumbai, Goa-based organizations-Cooj, Chennai-based organization-Sneha Foundation and Bengaluru-based Parivarthan Counselling, Training and Research Centre [23].

The leading cause of suicide among HCPs in India, as previously discussed, is job-related stress. This includes academic anxiety in medical and nursing students; work-related stress in resident doctors, nurses and hospital assistants; and career stress among young professionals and senior consultants. Reduction in work-related stress can be achieved with measures such as the regularization of fixed-duty shifts and work hours, adequate breaks, stress management workshops and social and recreational activities. Additionally, a prompt addressal of complaints of harassment by a neutral authority or designated body can reduce the stress levels.

Regular health check-ups of medical/nursing students, healthcare professionals, and hospital employees should be implemented. Awareness programs targeting mental health and warning signs of suicides should be a part of the curriculum. Additional measures include promoting a healthy lifestyle and abstinence from addictive substances via audio-visual clips, graphic posters, or dedicated workshops. The above preventive measures have been suggested as per routine clinical practice but need to be evaluated in rigorous clinical studies.

## 3. Limitations

This paper has some limitations. There are many areas in the topic where we have added points based on our personal clinical experience, and studies in many areas of the topic still need to be carried out. There are many facets of research in this area across countries that need to be carried out.

## 4. Conclusions

Being associated with the healthcare sector has not rendered any form of immunity to HCPs regarding suicide. The opposite has been observed in studies. It is essential to quantify the magnitude of this problem among various sub-groups of HCPs viz. medical/nursing students and trainees, young professionals, consultants, ancillary medical staff, and hospital assistants before corrective changes can be made at each level. Students and trainees often resort to suicide due to academic and work-related stress, mental illness, and harassment. The administrations of healthcare institutions can adopt several preventive measures such as mental-health screening at regular intervals, stress-management training, harassment vigilance, raising awareness and stigma reduction around mental illness. Corrective measures such as the regularization of work hours, an adequate break between duty shifts to allow for good rest, nutrition, exercise and time to socialize and unwind should be adopted as well. The scientific literature on mental health problems and suicide among HCPs, such as review articles, case–control studies and verbal autopsies, is sparse. It has not received as much focus as farmer suicides in India. Extensive multi-centered studies evaluating the prevalence of mental-health problems, including suicidality among various subgroups of HCPs in India, are required to correctly ascertain the magnitude of the problem and aid policymakers in introducing comprehensive suicide-prevention steps to ‘save the saviors’.

## Data Availability

Not applicable.
